# In and Out-Door Air

**Published:** 1856-09

**Authors:** 


### IN AND OUT-DOOR AIR.

If a small portion of the air of a crowded room is made to pass up through distilled water, a sediment is left, which contains various colored fibres of clothing; portions of hair, wool; bits of human skin, or scales, with a kind of fungus growth, with its particles of reproduction, which adhere wherever they strike or fall on wet surfaces, or bruises, or sore places, and grow wherever they adhere; there is also a small amount of sand and dirt, with great numbers of the various forms of animal life.

No wonder, then, that the blood is soon tainted and corrupted by making sitting apartments of our chambers, by spending hours in crowded assemblies, or stage coaches, or rail cars, where every breath we draw is a mouthful of monster life.

But if that room be emptied for a few hours, and a portion of its atmosphere be treated in the same way, nothing will be found but a little sand and dirt, a few fibres of wool and cotton, only a trace of fungus, but no animal life, and no bits of skin and hair, and scales of dead human matter.

If five times the amount of neighboring out-door air undergoes the same process, a fibre of wool or cotton is now and then found, a little sand and dirt, with specimens of fungus and their atoms of reproduction, but no traces of decayed animal matter, and no signs of organic life; thus showing, that in our close apartments we are surrounded with organic living bodies, and that animal matter living, dead and decayed, loads the atmosphere which we breathe in the chambers of our dwellings and crowded rooms, and that these corrupting particles are swallowed, and are breathed into the system every moment of in-door existence, thus strongly urging us, by all our love of pure blood and high health, to hurry from our chambers at the earliest moment in the morning, and to consider every hour of out-door breathing, a gain of life.

